# Sensitivity of Proton NMR Relaxation and Proton NMR Diffusion Measurements to Olive Oil Adulterations with Vegetable Oils

**DOI:** 10.1021/acs.jafc.1c00914

**Published:** 2021-05-20

**Authors:** Donatella Ancora, Jerneja Milavec, Anton Gradišek, Mario Cifelli, Ana Sepe, Tomaž Apih, Boštjan Zalar, Valentina Domenici

**Affiliations:** † Dipartimento di Chimica e Chimica Industriale, Università di Pisa, via Moruzzi, 3, 56124 Pisa, Italy; ‡ Department of Condensed Matter Physics, Jožef Stefan Institute, 39 Jamova Cesta, SI-1000, Ljubljana, Slovenia

**Keywords:** ^1^H NMR, *T*_1_, *T*_2_, diffusion, adulteration, DOSY, dynamics, olive oil, vegetable oil

## Abstract

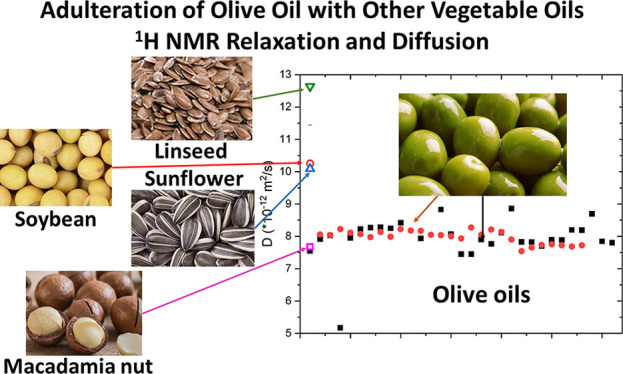

Olive oils and, in particular, extra-virgin olive oils (EVOOs) are one of the most frauded food. Among the different adulterations of EVOOs, the mixture of high-quality olive oils with vegetable oils is one of the most common in the market. The need for fast and cheap techniques able to detect extra-virgin olive oil adulterations was the main motivation for the present research work based on ^1^H NMR relaxation and diffusion measurements. In particular, the ^1^H NMR relaxation times, *T*_1_ and *T*_2_, measured at 2 and 100 MHz on about 60 EVOO samples produced in Italy are compared with those measured on four different vegetable oils, produced from macadamia nuts, linseeds, sunflower seeds, and soybeans. Self-diffusion coefficients on this set of olive oils and vegetable oil samples were measured by means of the ^1^H NMR diffusion ordered spectroscopy (DOSY) technique, showing that, except for the macadamia oil, other vegetable oils are characterized by an average diffusion coefficient sensibly different from extra-virgin olive oils. Preliminary tests based on both NMR relaxation and diffusometry methods indicate that eventual adulterations of EVOO with linseed oil and macadamia oil are the easiest and the most difficult frauds to be detected, respectively.

## Introduction

Extra-virgin olive oil (EVOO) represents one of the most important and nutritionally valuable edible oil and is a basic ingredient of the Mediterranean diet. As defined by the “International Olive Council”,^1^ EVOO is obtained by cold-pressed olives (*Olea europaea*), exclusively with mechanical methods, and it has a free acidity ≤0.80%, expressed as the percent of oleic acid. Free acidity is one of the main discriminants among different commercial categories of olive oils, which include virgin olive oil (VOO) and lampant olive oils, such as olive oil (OO), olive pomace oil (OPO), and refined olive oil (ROO). Lampant olive oils cannot be directly edible, which is why they need to be further processed and mixed with virgin olive oil before consumption.^1^ Due to low availability of EVOOs with respect to the market demand and, consequently, due to EVOOs’ high cost, they are a frequent target of counterfeit practices. Fortunately, several types of olive oil frauds can be easily detected using standard and regular analytical methods.^2^ Adulterations of EVOOs include replacing of parts of EVOO constituents or alteration of the proportional quantity of one or more of EVOO’s chemical components. In most cases, adulterations are performed by preparing mixtures of olive oils of different categories, refined olive oils, and/or other vegetable oils.^3,4^ In recent years, very sophisticated adulterations, based on the addition of soft refined oils, soft deodorized, or soft deacidified virgin olive oils were developed, which cannot be discovered by using standard analytical methods.^5^ However, the addition of edible oils produced from seeds (such as sunflower seed and linseed oils), legumes (such as peanut and soybean oils), and nuts (such as walnut and macadamia oils) still remains a very frequent mean of adulteration of EVOOs.

Among different analytical techniques developed to identify EVOO’s adulterations,^2^ a first type is centered on the identification and quantification of specific chemical markers, such as some polar components, campesterols, or tocophenols, by means of gas or liquid chromomatographic techniques coupled with different high-resolution detectors. A second class of techniques adopts instrumental methods to investigate a large number of chemical constituents and identify specific spectral profiles distinctive of EVOOs. As reported in a recent comprehensive review about this topic,^2^ spectroscopic methods such as infrared (Fourier transform-infrared (FT-IR), mid-infrared (MIR), and near infrared (NIR))^4^ and Raman^6^ spectroscopy, ultraviolet–visible (UV–vis) absorption^7^, and fluorescence spectroscopy,^8^ and nuclear magnetic resonance (NMR) spectroscopy^9,10^ have had tremendous developments in the last 10 years due to the possibility to perform a relatively cheap, rapid, and nondestructive analysis of EVOOs.^11,12^

High-resolution NMR techniques based on the acquisition of proton, carbon-13, and phosphorus-31 NMR spectra^13−16^ have been widely applied for the characterization of different chemical components of olive oils and other vegetable oils. In a recent work,^16^^1^H NMR spectral features of different edible oils produced from Brazil nut, linseed, sesame (toasted and raw), and soybean have been analyzed in terms of different percentages among oleic, palmitic, linoleic, and linolenic acids, which affect the relative intensities of ^1^H NMR signals between 1 and 3 ppm. The analysis of ^1^H NMR spectral features in combination with statistical multivariate methods^17^ was successfully used to discriminate EVOOs produced in different geographic areas^18,19^ or from different botanic cultivars.^20^ Few examples of high-resolution ^1^H NMR studies aimed to detect olive oil adulterations have been published.^21^

Other ^1^H NMR techniques, such as ^1^H NMR fast field cycling relaxometry,^22−29^ time domain and low-field ^1^H NMR relaxometry,^30−33^ and ^1^H NMR diffusometry^27,34^ have been applied to characterize oils of different origins and to detect eventual adulterations in olive oils.

The measure of ^1^H NMR relaxation times, longitudinal (*T*_1_) and transverse (*T*_2_),^35^ at different Larmor frequencies can give indirect information about several chemical and physical properties of edible oils having a variable fatty acids’ composition, and it has been used to study the effect of thermal oxidation and desiccation processes.^22,23,26,32^ The analysis of ^1^H NMR *T*_1_ dispersions of EVOOs and the analysis of *T*_1_ at low magnetic fields have been used to investigate supramolecular structural features, such as the occurrence of inverse-micelle-like organization of triglycerides in extra-virgin olive oils,^22^ and dynamic information, such as correlation times associated with rotational motions and self-diffusion constants.^23−25,27^ A low-field (LF) ^1^H NMR relaxation method based on the reconstruction of 2D and 3D plots to correlate *T*_1_ and *T*_2_ distributions has been applied to check the thermal oxidation of linseed oils^36^ and to detect several types of adulterations of vegetable oils.^37,38^ This rapid and relatively cheap LF NMR relaxation method seems particularly useful to study the effect of oxidation in several vegetable oils, such as the macadamia,^39,40^ linseed,^41^ sunflower^42,43^ and other blended oils,^44,45^ which are used not only for consumption but also for painting, energy, and biomass applications.

Few works have been published about the measurements of diffusion coefficient using high-gradient diffusion NMR techniques on olive oils. ^1^H diffusion ordered spectroscopy (DOSY) NMR was used in a few explorative works to demonstrate the suitability of a direct method for discrimination of several types of adulterations of olive oils with several vegetable oils, such as sunflower, soybean, hazelnut, and peanut oil.^27,34,46^

In this paper, we report an original study based on ^1^H NMR relaxation and ^1^H NMR diffusion measurements applied to several vegetable oils, namely, soybean (SoO), macadamia nut (MO), linseed (LO), sunflower (SuO), and about 60 extra-virgin olive (EVOO) oils produced in Italy in two different regions, Apulia and Tuscany. In particular, a comparison between EVOO and vegetable oil samples in terms of the relaxation times (*T*_1_ and *T*_2_) measured at low resolution NMR setups (i.e., 2 and 100 MHz) and of the average diffusion coefficient (*D*) measured by means of the DOSY ^1^H NMR technique, is reported and discussed in view of the applicability of these NMR methods in discriminating several adulterations of EVOOs with vegetable oils.

## Materials and Methods

### Oil Samples

In this work, we have selected 59 EVOOs produced in Tuscany and in Apulia from a larger set,^24,25^ whose details about the harvesting year, cultivars, and geographic area of olive trees production are reported in the Supporting Information (Table S1). The EVOO samples are labeled as “*at_X*” and “*ap_Y*” to indicate whether they are from Tuscany or from Apulia, respectively, where *X* and *Y* are the consecutive numbers. Vegetable oils produced from soybeans (SoO), linseeds (LO), macadamia nuts (MO), and sunflower seeds (SuO) were purchased at a local store. All oil samples were stored in dark conditions, in 25 mL dark glass bottles, at a temperature ≤5 °C.

### NMR Methods

^1^H NMR relaxation measurements on oil samples were performed using different NMR spectrometers working at ^1^H Larmor frequencies of 2 and 100 MHz.

A rock core analyzer spectrometer (Magritek, https://magritek.com/) operating at the ^1^H Larmor frequency of 2 MHz was used to determine the proton spin–lattice relaxation times, *T*_1_, and proton spin–spin relaxation times, *T*_2_. This instrument is a wide-bore (diameter = 55 mm) NMR system, using a permanent magnet, working at low resolution, specifically for soft and solid matter (it was originally developed to measure the porosity of concrete or the oil content in rocks). About 20 mL of oils were transferred to weighing bottles (diameter = 30 mm, *V* = 20 mL) and put into the bore at room temperature with temperature control of ±0.5 °C. The inversion recovery sequence was used for *T*_1_ measurements, with τ (variable time delay) ranging from 1 ms to 1 s in 20 steps, using a 20 μs π/2 pulse (90°). The number of scans (NS) was 4 per sequence and the repetition time (RT) was equal to 3 s. The Carr–Purcell–Meiboom–Gill (CPMG) sequence^47,48^ was used for the *T*_2_ measurements. The τ (time delay) was 200 μs and 1000 echos were used. The π/2 pulse was 40 μs. NS was 16 and RT was 0.5 s.

Measurements of ^1^H NMR relaxation times *T*_1_ and *T*_2_ were also performed using a horizontal bore Oxford magnet operating at 100 MHz. Temperature was controlled by a gas flow system, and the temperature control was ±0.5 °C. About 2 mL of oil were transferred to MRI glass tubes (diameter = 0.5 cm, *h* = 1.5 cm) and put into the probe. The inversion recovery sequence used for *T*_1_ measurements, the π/2 pulse was 3.5 μs, τ varied from 0.2 ms to 3 s in 21 steps. NS was 2 and RT was 3 s. The spin echo sequence was used for *T*_2_ measurements. The time delay τ varied from 0.02 ms to 2 s in 12 steps, π/2 pulse was 3.5 μs, NS was 2, with RT equal to 3 s. Temperature control of ±0.1 °C was used.

Pulsed gradient stimulated echo (PG-STE) diffusion ^1^H NMR measurements were carried out on an Ultrashield Advance III Bruker spectrometer operating at a proton Larmor frequency of 500 MHz, equipped with a 5 mm diffusion probe yielding a maximum *Z* gradient of 74 G/cm, that was continuously cooled by flowing water at room temperature. All the oil samples were transferred into 1 mm NMR glass tubes, covered with Teflon tape and placed into a 5 mm NMR tube probe at room temperature. Diffusion spectra were obtained using a PG-STE sequence with a gradient pulse δ of 10 ms, diffusion time Δ of 30 ms, and gradient amplitude *g* ranging from 1 to 74 G/cm in 16 increments. The ^1^H DOSY NMR technique^49^ was used to record a pseudo-2D spectrum for each oil sample (see Figures S1 and S2 in the Supporting Information). Diffusion coefficients, *D*, were calculated by fitting the monoexponential dependence of attenuation of signal intensity, *I*, on the gradient amplitude according to eq 1:
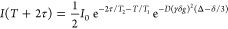
1where *T* is the storage time between π/2 pulses, τ is the echo delay, *T*_1_ and *T*_2_ are the longitudinal and transverse relaxation times, respectively. As reported in the Supporting Information, the ^1^H DOSY NMR methodology allows us to obtain a 2D plot showing a two-dimensional peak for each ^1^H NMR signal in the monodimensional spectrum. From the center of each cross-peak in the 2D plot, the values of diffusion coefficients can be extracted, one for each ^1^H signal. In the following data analysis, for each oil sample, we are reporting the value *D* obtained from the average over the diffusion coefficients measured from different ^1^H NMR signals, as described in the Supporting Information. Repeatability of the measurements of the diffusion coefficients was ensured on a representative EVOO sample (label “*ap_18*”). The relative error on the average value of *D* was evaluate to be less than 5%, as obtained from measurements repeated in triplicate and at different times of the day, in order to check the effect of the external temperature variability.

### Spectral and Data Analysis

NMR data were analyzed using integration of the spectra. In the case of the high-resolution NMR spectra at 100 MHz, parts of the spectra were integrated to obtain the monoexponential spin–lattice or spin–spin relaxation times, as reported in ref (25). In the case of the low-resolution NMR spectra at 2 MHz, the entire broad spectra were integrated and the relaxation times were obtained using a two-component relaxation model.^25^ Diffusion coefficients were calculated as described in the previous section by using the Bruker Topspin 2.0 software.

## Results and Discussion

### ^1^H NMR Spectra of Different Vegetable Oils

**Figure 1:**
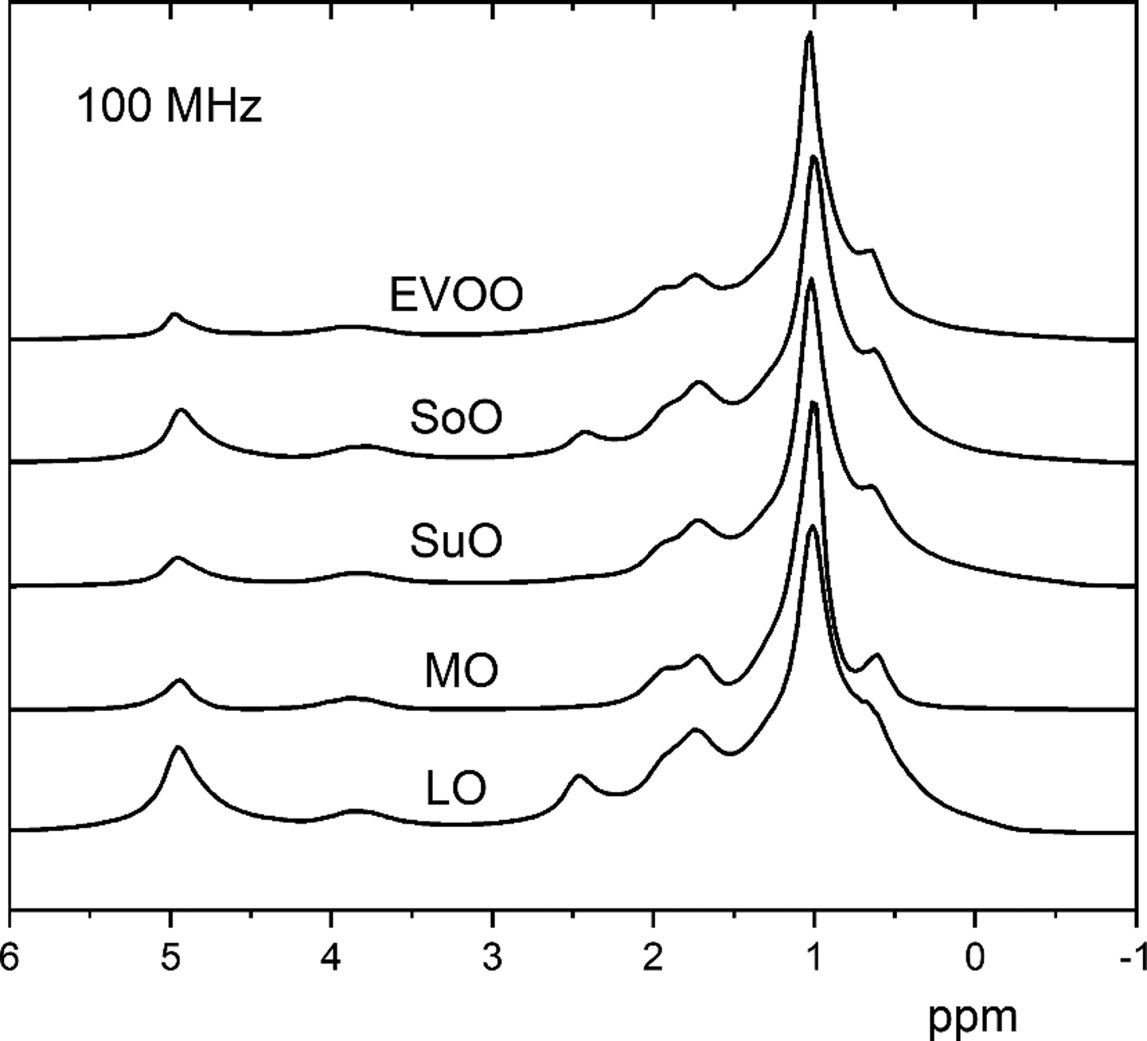
^1^H NMR spectra of a representative EVOO sample (at_28) and other vegetable oils (soybean, sunflower, macadamia, linseed), recorded at the 100 MHz magnet, at room temperature. The spectra have been normalized to the strongest peak each. Possible slight frequency shifts are due to the field inhomogeneities due to the sample size.

Figure 1 shows the ^1^H NMR spectra of a representative EVOO sample (*at_28*) and other vegetable oils, namely, soybean oil (SoO), sunflower oil (SuO), macadamia oil (MO), and linseed oil (LO), recorded at room temperature, at a Larmor frequency of 100 MHz. Due to the relatively inhomogeneous magnetic field, some of the peaks corresponding to different proton species in the oil samples are broad and partially overlapped.^25^ It is interesting to note some differences among the ^1^H NMR spectra of vegetable oils with respect to the EVOO sample (*at_28*). For instance, the ^1^H NMR spectrum of LO (Figure 1) shows an intense peak centered at ∼2.5 ppm and a quite intense peak centered at 5 ppm. The latter peak is usually attributed to the olefinic protons of all unsaturated fatty acids, while the signal at 2.5 ppm is related to the amount of linolenic and linoleic acids present in linseed oil in higher amounts than in olive oil.^17,34^ Other seed oils, i.e., SuO, MO, and SoO, show the peak at 2.5 ppm as well, but with lower intensity. As also reported in refs (34) and (50), the intensity of these two peaks is higher in soybean and sunflower oils with respect to EVOOs, and this is one of the reasons of the increasing interest in NMR techniques for rapid screening of adulterations of olive oils with vegetable oils. As a general remark, the relative intensities of the main peaks observed at 100 MHz are related to different percentages among fatty acids in different types of vegetable oils. Interestingly, this can be observed not only in high-resolution ^1^H NMR spectra at a high magnetic field^13,14,16,17^ but also in relatively low-resolution ^1^H NMR spectra, as those reported in Figure 1.

### ^1^H NMR Relaxation Measurements at 2 and 100 MHz

**Figure 2:**
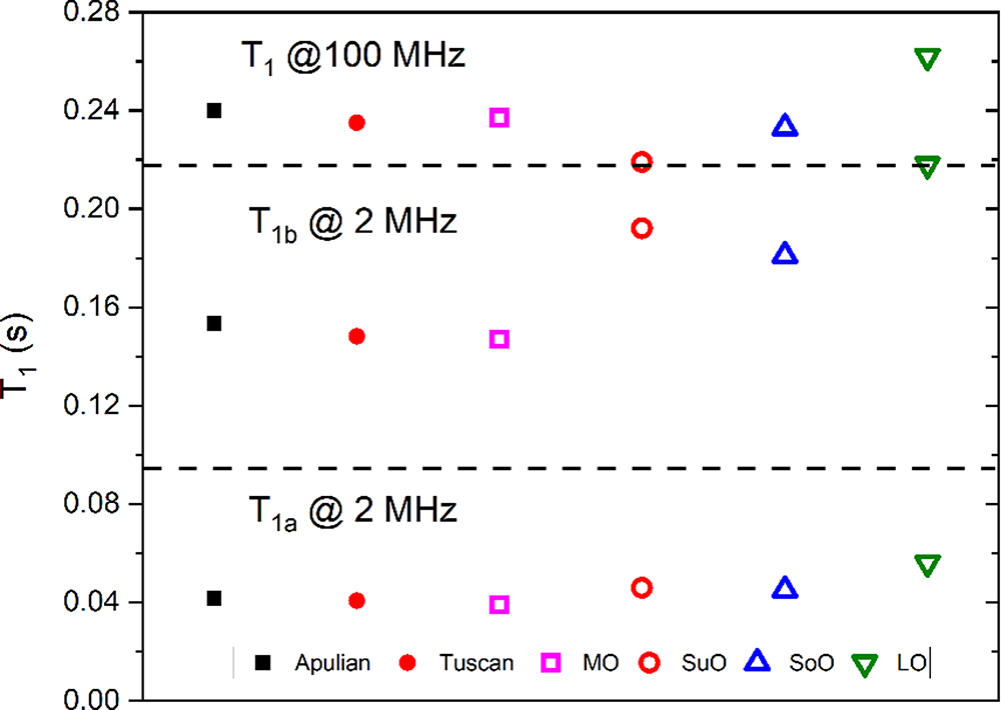
Spin–lattice relaxation times measured on a set of oils at two different setups. Points for Apulian and Tuscan EVOOs are the average values over a large number of samples while those for other oils belong to a single sample each. Horizontal dashed lines were added for easier interpretability of the figure.

**Figure 3:**
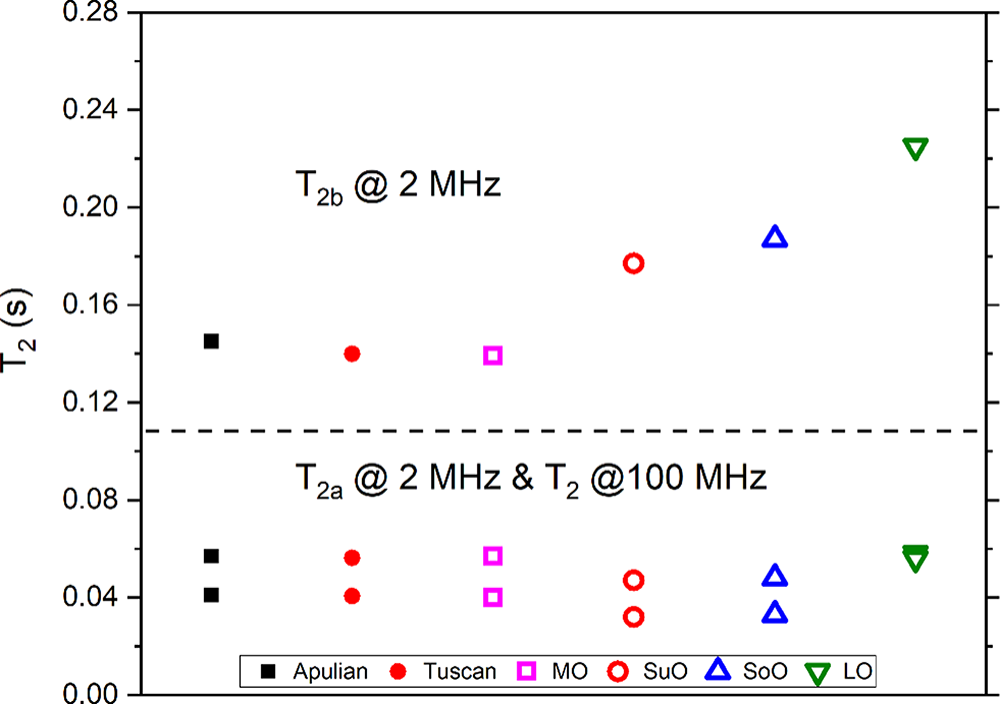
Spin–spin relaxation times measured on a set of oils at two different setups. Points for Apulian and Tuscan EVOOs are the average values over a large number of samples, while those for other oils belong to a single sample each. A horizontal dashed line was added for easier interpretability of the figure.

As reported in previous works,^24,25^ both spin–lattice and spin–spin relaxation processes in oils are clearly not monoexponential at 2 MHz; instead they can be satisfactorily described using a two-components relaxation model, with the two components labeled as *T*_1a_ and *T*_1b_ and *T*_2a_ and *T*_2b_, respectively. In the relaxation data analysis,^24,25^ the ratio of the two components’ amplitudes, both for the longitudinal and transverse relaxation processes, was left as a free parameter, and it turns out that the weights of components obtained were typically around 2:1 for the long component.^25^ The two components were attributed to the more rigid (shorter component) and more flexible parts (longer component) of the trygliceride molecules, as also reported in previous relaxation NMR studies on olive oils^25,36^ and, similarly, on pistachio oils.^23^ Relaxation measurements performed at 100 MHz allowed us to obtain the relaxation times, *T*_1_ and *T*_2_, for different proton species, by integrating the corresponding areas of the proton NMR spectra of the oil samples. In this case, both the longitudinal and transverse relaxation processes are monoexponential. For simplicity, for each oil sample, the relaxation times, *T*_1_ and *T*_2_, obtained on the strongest peak in the ^1^H NMR spectrum recorded at 100 MHz^24,25^ are reported here. Figures 2 and 3 show the comparison between *T*_1_ and *T*_2_ values measured at 2 MHz (i.e., two components *T*_1a_ and *T*_1b_ in Figure 2 and two components *T*_2a_ and *T*_2b_ in Figure 3) and at 100 MHz (single average values, *T*_1_ and *T*_2_) for each oil. As reported in a previous work,^25^ the percentage error on the experimental relaxation data was evaluated from measurements in triplicate and it ranges from a minimum of 1% to a maximum of 8%. Similarly, the error associated with the values of *T*_1_ and *T*_2_ in vegetable oil samples is in the range of 1–5%. For an easier comparison, the values for *T*_1_ and *T*_2_ are plotted on the same vertical scale. Moreover, Figures 2 and 3 show the *average* values of relaxation times measured in a large set of EVOO samples produced in two Italian regions, namely, Apulian and Tuscan EVOOs, since a detailed analysis of the variability and common features of this large set of EVOO samples was presented in a previous work.^25^ The main purpose here is to compare the average values of relaxation times obtained on EVOO samples with those obtained on other vegetable oils. From Figure 2, it can be seen that the *T*_1_ values for EVOOs and macadamia oil (MO) are very similar at both values of magnetic fields, while the other three oils have substantially different values, especially the linseed oil (LO), whose values of *T*_1_ are all longer than in other oils.

Similar features are observed for *T*_2_ values reported in Figure 3. Here, the macadamia oil sample has very similar values of *T*_2_ to those of EVOOs, while the major differences between vegetable oils and EVOOs is observed for the *T*_2b_ component measured at 2 MHz. In particular, sunflower, soybean, and linseed oils have longer values of *T*_2b_ with respect to EVOO and MO samples. Moreover, the data reported in Figure 3 show that for EVOO, MO, and SuO oil samples *T*_2a_ < *T*_2_ at 100 MHz, while for the SoO and LO samples, the opposite holds.

As a general remark, while the *T*_1_ values at 100 MHz are longer than either of the two components at 2 MHz, the *T*_2_ values at 100 MHz are closer to the *T*_1a_ component at 2 MHz. This is reasonable, since spin–lattice NMR relaxation is known to have a strong field-dependence, whereas *T*_2_ is only weakly dependent on the field. As observed in other works,^23,27,32^ the sensitivity of relaxation times measured at different magnetic fields to different types of oils is promising for the detection of adulterations in EVOOs. In particular, relaxation data recorded at 2 MHz indicate that the values of *T*_1_ and *T*_2_ obtained in linseed, sunflower, and soybean oils are very different from those measured in EVOOs. For instance, component b of *T*_1_ (at 2 MHz) of LO, SuO, and SoO samples are, respectively, 41%, 30%, and 35% larger than the average values of EVOOs. Similarly, component b of *T*_2_ (at 2 MHz) of LO, SuO, and SoO samples are, respectively, 46%, 35%, and 23% larger than the average values of EVOOs. Based on these results, future works will be a focus on a large set of vegetable oils in order to confirm these findings.

### Diffusion ^1^H NMR Measurements

Following the approach proposed by Šmejkalová et al.,^34^^1^H NMR spectra of a large set of extra-virgin olive oil and vegetable oil samples were acquired at 500 MHz without any sample preparation. 2D plots recorded by means of the DOSY ^1^H NMR technique^49^ were analyzed in terms of the diffusion coefficients corresponding to different peaks (see the Supporting Information for details). For simplicity, for each oil sample, we are reporting the average value among the diffusion coefficients measured for each crosspeak in the DOSY ^1^H NMR 2D plot, which corresponds to different ^1^H signal attributed to different parts of the triglycerides and fatty acids, similarly to what was reported in a previous work.^34^

The representative EVOO sample *ap_18* was analyzed four times, in triplicate, at different hours of the day to ensure the reproducibility of the measurements, in view of potential temperature variations during the day. The average value of diffusion for the reference EVOO sample was *D* = (8.2 ± 0.3) × 10^–12^ m^2^/s. The low standard deviation value obtained indicates a good repeatability of the method. Moreover, the reproducibility of diffusion NMR measurements and several tests regarding the temperature-dependence of diffusion coefficients in several EVOO samples were performed (see the Supporting Information), showing the reliability and sensitivity of the technique.

**Figure 4:**
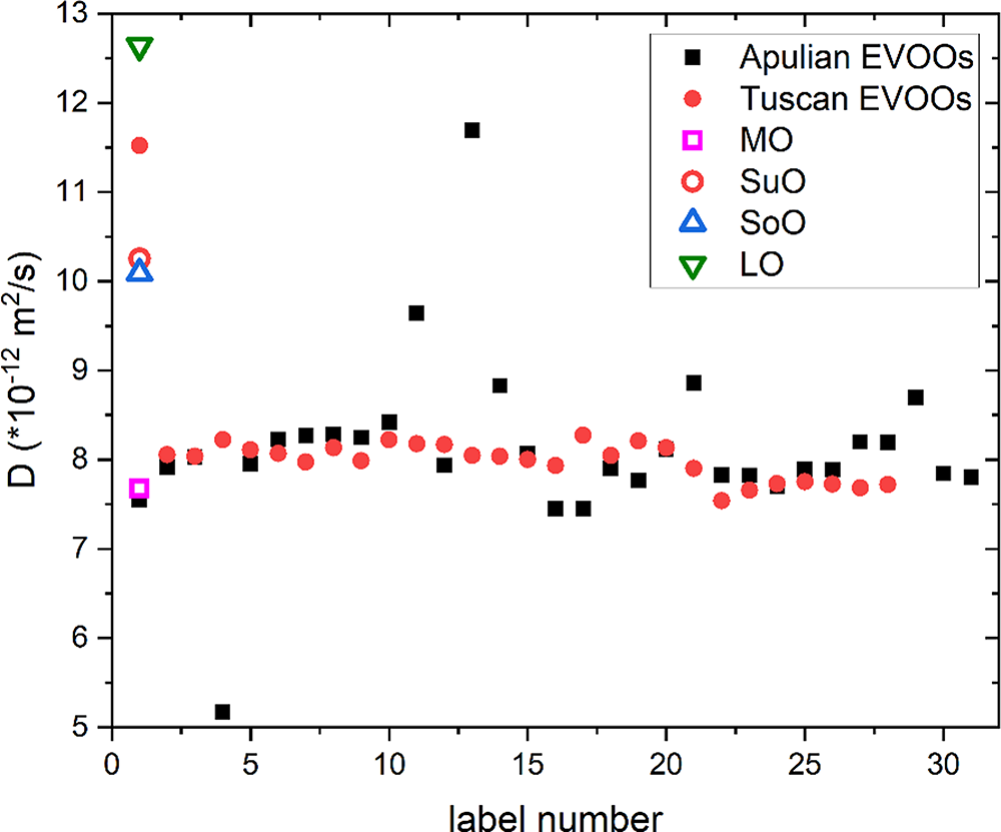
Average diffusion constant values measured by the ^1^H NMR DOSY technique on a set of extra virgin olive oils, namely, Apulian and Tuscan EVOOs, and vegetable oils from macadamia (MO), sunflower (SuO), soybean (SoO), and linseed (LO) oils.

Figure 4 shows the diffusion contant values, *D*, recorded at room temperature (namely *T* = 298 K) for 59 EVOO samples produced in different regions of Italy, namely, Apulia and Tuscany, as well as those of macadamia, sunflower, soybean, and linseed oils. Based on our knowledge, this is the first time that this methodology was applied to a large set of EVOOs. The Tuscan EVOOs have rather similar average values of *D*, around 8.1 ± 0.7 × 10^–12^ m^2^/s. On the other hand, the values of *D* of the Apulian EVOOs are more scattered (with a total average value of 8.2 ± 1.0 × 10^–12^ m^2^/s). These variations could be explained in terms of variability of the fatty acids components (i.e., percentage of oleic acid, ratio between oleic and linoleic acids) in Apulian EVOOs, as also reported in a previous work based on high-resolution ^1^H NMR spectroscopy.^51^ In both EVOO sets, there are few outliers with the values of *D* significantly different from the average value, namely, from ∼5 to ∼12 × 10^–12^ m^2^/s. Concerning the EVOO samples, the values of self-diffusion here reported on a relatively large set of Italian EVOO samples are in line with those obtained on extra virgin olive oils from pulse gradient spin echo (PGSE) ^1^H NMR and ^1^H NMR relaxometry methods.^27,28^ As it can be seen in Figure 4, the macadamia oil (MO) has a similar average diffusion constant than EVOOs, while the other three vegetable oils have considerably larger values than olive oils. In particular, the average diffusion coefficient of LoO, SuO, and SoO are, respectively, 53%, 22%, and 20% larger than the average value found for the EVOO samples. Data obtained for soybean and sunflower oils are in line with those reported in ref (34). Similarly to the results of relaxation measurements reported in the previous section, even in the case of diffusion measurements, linseed oil shows major differences with respect to EVOOs.

### EVOOs’ Adulterations: Relaxation and Diffusion NMR Measurements

Up to this point, we have reported the relaxation and diffusion NMR properties of individual oil samples. The motivation for the second part of this study was to investigate whether the mixing of a vegetable oil to olive oil samples changes the relaxation and diffusion properties of the EVOOs enough so that they could be distinguished from pure EVOOs. Here, we consider two aspects in order to make the adulteration of EVOOs “profitable”: (i) the vegetable oil used for adulteration should be considerably cheaper than the EVOO and (ii) the amount of vegetable oil added should be substantial (not in traces). The first aspect leaves out adulteration with macadamia oil which, even if the relaxation data and the diffusion coefficient are close to the values obtained in EVOOs, is quite expensive, thus making it an unreasonable adulterant. On the other hand, as shown in a recent work,^39^ macadamia oil has attracted a major interest in the field of biodiesel and other fuels derived from vegetables and plants which may lead to a price drop in the future. On the contrary, sunflower, soybean, and linseed oils are cheaper and have been used in several frauds of extra-virgin olive oils.^34,38,41,46,50^

**Figure 5:**
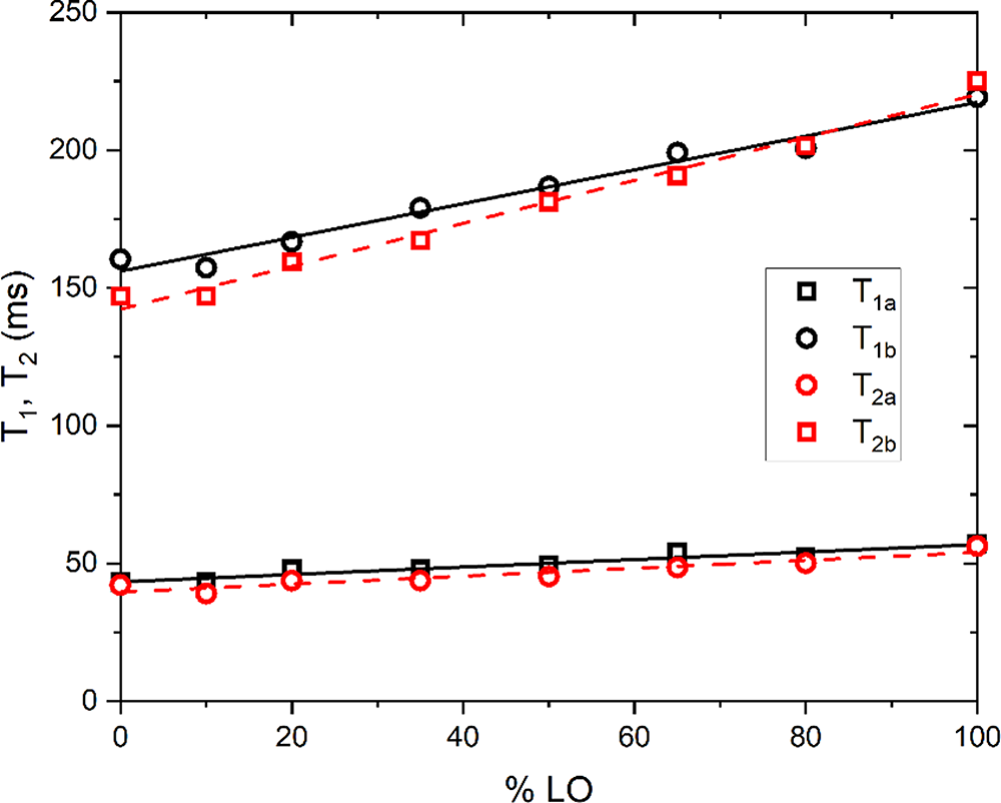
Proton spin–lattice and spin–spin relaxation times measured at 2 MHz at room temperature for a mixture of EVOO sample labeled *at_28* and linseed oil, expressed in volume percent of LO added. Solid black and dashed red lines are the linear fits to the *T*_1_ and *T*_2_ data and they should serve as a guide to an eye.

In the first experiment, we mixed the EVOO sample *at_28* with linseed oil (LO) in different volume ratios, and the longitudinal and transverse ^1^H NMR relaxation times were measured at 2 MHz. Figure 5 shows the dependence of *T*_1_ and *T*_2_ values (both showing two components, a and b) as a function of the volume percent of added linseed oil. As seen from the figure, relaxation times get longer with the amount of linseed oil added to the EVOO sample, and the trend is roughly linear with the percent of added adulterant. In particular, we can note that the slope of the linear trend of *T*_1b_ and *T*_2b_ is large enough to observe a relaxation time variation of ∼10% already when a low percentage (∼10%) of linseed oil is added to extra-virgin olive oil. Similar values are reported in the literature where percentages ranging between 5 and 20% of several vegetable oils used as EVOO adulterants have been successfully detected by means of low-field NMR techniques,^2,46^^1^H NMR and FT-IR spectroscopy,^4,52,53^ coupled with multivariate statistical approaches.

**Figure 6:**
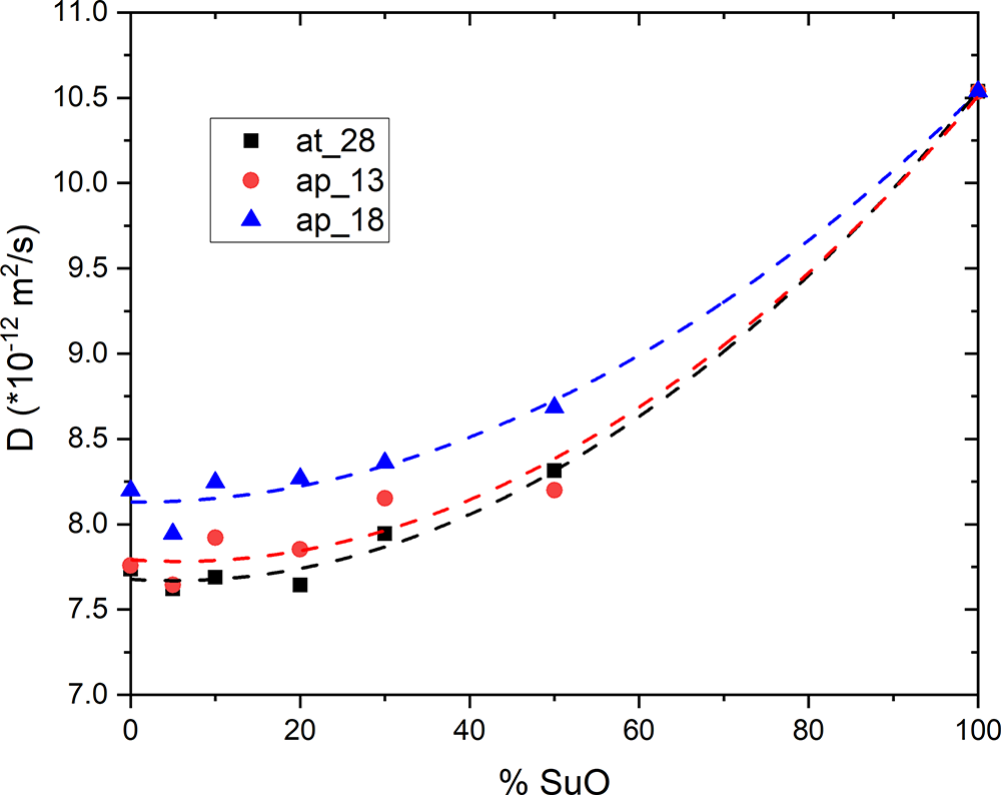
Diffusion constant *D* measured by ^1^H NMR DOSY of a mixture of EVOO and sunflower oils as a function of the added volume percent of SuO. Three different EVOO samples have been used for this test as reported in the text. Dashed lines are fits to the data using a quadratic function and should serve as a guide to the eye.

In the second experiment reported here, we focused on the NMR diffusion measurements. Sunflower oil was chosen as an adulterant instead of linseed oil, as the diffusion constant of the latter was the largest of all samples, while the value of *D* measured for the sunflower oil was closer to the average value of the two sets of Italian EVOOs. In this case, different amounts of sunflower oil were added (in volumetric percent) to three different EVOOs, labeled as *at_28*, *ap_13*, and *ap_18*. Figure 6 shows the dependence of the diffusion constant on the volume percent of added sunflower oil. As seen from the figure, the diffusion coefficient increases by increasing the percentage of the sunflower oil added, with a nonlinear function. The nonlinearity of the trend of diffusion constant as a function of the volume percentage of adulterant oil added is not surprising, since a number of parameters influencing the average value of the diffusion coefficient are not linear, such as viscosity.^27^ Similar behavior has been observed in other binary mixtures^54^ and is also known from the literature for systems forming microemulsions, which oils are. Moreover, it should be noted that the addition of different oils, having different viscosities for instance, led to mixtures which are not necessarily homogeneous. In this preliminary test, for examples, we noted the presence of heterogeneities due to nonperfect mixing, which can explain the nonlinearity as well. This aspect is probably one of the main limitation of the applicability of the method and it will be further investigated in the future. Moreover, as also observed in refs (24 and 55), the diffusion coefficient is strongly temperature-dependent, which is another aspect to take into account: the temperature should be carefully monitored during the measurements. Temperature dependencies of *D* for two representative oil samples, an EVOO and SuO, are shown in the Supporting Information (see Figure S3). Nevertheless, to the best of our knowledge, this is the first study where vegetable oils were investigated by means of the ^1^H NMR DOSY technique in order to detect adulterations of extra-virgin olive oils. Future studies about the reliability of diffusion measurements to detect olive oil adulterations are in progress in order to produce a robust protocol for rapid applicative uses. Moreover, the possibility to measure *T*_1_, *T*_2_, and *D* on a single instrument by using low-field, low-resolution NMR setups will be explored.^56^

To conclude, in this work the ^1^H NMR relaxation times, *T*_1_ and *T*_2_, of several vegetable oils (macadamia, linseed, soybean, and sunflower oils) were measured with low-resolution NMR systems, working at 2 and 100 MHz. These values were compared with those obtained on a large set of extra-virgin olive oil samples produced in Italy, reported in another study.^25^ The aim of the comparison among the relaxation times here reported was the discussion of the potentialities of NMR relaxation times to discriminate among different vegetable and olive oils. Results reported here show that macadamia oil has a very similar relaxation behavior than EVOOs, while the values for the linseed oil differ the most. A case study of mixing different volume percentages of linseed oil with a reference EVOO sample showed a linear relationship between the values of *T*_1_ and *T*_2_ measured at 2 MHz and the added amount of adulterant, which is in line with other spectroscopic methods developed in view of detections of adulterants. In this work, ^1^H NMR DOSY experiment was applied to measure the average self-diffusion coefficient, *D*, of about 60 EVOO and 4 vegetable oil samples. The comparison among self-diffusion coefficients of different oil samples showed that (in a similar fashion than the relaxation times) the macadamia oil exhibits very similar diffusion properties than most EVOOs. This likely reflects the very similar fatty acid and triglycerides composition of macadamia and olive oils, while the main differences observed with other vegetable oils can be explained in terms of fatty acids relative percentages, in particular, related to the amount of oleic/linoleic and linolenic acids. Similarly, self-diffusion coefficients of sunflower, linseed, and soybean vegetable oils are significantly larger than those of typical EVOOs. A proof-of-concept adulteration experiment was performed by mixing different volume percentages of sunflower oil to EVOOs, showing that the trend of the diffusion coefficient as a function of volume percentage of SuO added is monotonous but not linear. As discussed above, this aspect could represent a limitation for the applicability of the method; however, further investigations are in progress in order to rationalize this behavior.

Our results indicate that the analysis of *T*_1_, *T*_2_, and *D* is promising in detecting EVOOs adulterated with other vegetable oils. A practical application would instead use a tabletop setup to measure all three parameters (note that our version of 2 MHz rock core analyzer does not allow diffusion measurements). Tabletop systems that can measure all three parameters are already available, either in a prototype form or as several commercially available NMR mouse systems. A further improvement in detection of adulteration would be brought forward with the use of multidimensional data, by means of clustering or discriminant analysis. Both the tabletop application and multidimensional data analysis fall within the scope of future work.

## Supporting Information Available

List of oil samples investigated in this work, ^1^H NMR DOSY experimental details and text about temperature dependence of diffusion coefficients (PDF)

## Notes

^§^ D.A. is a former student at Dipartimento di Chimica e Chimica Industriale, Università di Pisa.

## Notes

^#^ J.M. is a former Ph.D. student at Jožef Stefan Institute.
