# Supplementary material for: Sensitivity of Proton NMR Relaxation and Proton NMR Diffusion Measurements to Olive Oil Adulterations with Vegetable Oils
Source: J Agric Food Chem. 2021 May 20;69(41):12081–8. doi: 10.1021/acs.jafc.1c00914 (PMC8532151; doi:10.1021/acs.jafc.1c00914)
Supplement: Supplementary file 1 — jf1c00914_si_001.pdf [file jf1c00914_si_001.pdf]

Sensitivity of Proton NMR Relaxation and Proton NMR Diffusion Measurements to Olive Oil Adulterations with Vegetable Oils

Donatella Ancora[1§], Jerneja Milavec[2#], Anton Gradišek[2], Mario Cifelli[1],*

Ana Sepe[2], Tomaž Apih[2], Boštjan Zalar[2] and Valentina Domenici[1]*

1. Dipartimento di Chimica e Chimica Industriale, Università di Pisa, via Moruzzi, 3 – 56124 Pisa (Italy);

2. Department of Condensed Matter Physics, Jožef Stefan Institute, 39 Jamova Cesta, SI-1000, Ljubljana (Slovenia).

[§] former student at Dipartimento di Chimica e Chimica Industriale.

[#] former Ph.D. student at Jozef Stefan Institute.

* Corresponding authors.

E-mail: valentina.domenici@unipi.it & anton.gradisek@ijs.si

SUPPORTING INFORMATION

Table S1: List of oil samples investigated in this work. Producer, Geographical area, Cultivar, harvesting year and additional information are indicated for each sample, with the label and number used in the data analysis.

Sample	Producer	Area	Cultivar	Year	Further information	Label for Figures
**at_1**	Agriturismo Pane&Vino	Gabbro (LI)	Blend	2012	biologic oil	1
**at_2**	Agriturismo Cappellese	Castelnuovo della Misericordia (LI)	Frantoio, Leccino, Moraiolo	2012	biologic oil	2
**at_3**	Azienda Agrituristica Casolar de No' altri	Larciano (PT)	Blend	2010	biologic oil	3
**at_4**	Azienda Orzalesi	Rosignano Marittimo (LI)	Frantoio, Leccino, Moraiolo	2012		4
**at_5**	Azienda Agricola "Le Ceppite"	Rosignano Marittimo (LI)	Frantoio, Leccino, Moraiolo	2012		5
**at_6**	Azienda Agricola "Antica Fonte"	Rosignano Marittimo (LI)	Frantoio, Leccino, Moraiolo	2012		6
**at_7**	Azienda Agricola "Esposito Susanna et Atria"	Bibbona (LI)	Frantoio, Leccino, Moraiolo	2012		7

**at_8**	Franci Frantoio IGP	Montenero D'orcia	Frantoio, Leccino, Moraiolo	2012		8
**at_9**	Oliveto Fonte di Foiano	Castagneto Carducci	Frantoio, Leccino, Moraiolo	2012		9
**at_10**	Azienda Agricola Giovani	San Lorenzo, Suvereto	Frantoio, Leccino, Moraiolo	2012		10
**at_11b**	Tenuta "La Pineta"	Catiglion Fibocchi (AR)	Frantoio, Leccino, Moraiolo	2012	Filtered oil	11
**at_12**	Tenuta "S.Jacopo"	Cavriglia (AR)	Frantoio, Leccino, Moraiolo	2012		12
**at_13**	Azienda Agricola Cristiana Ruschi	Calci (PI)	Frantoio, Leccino, Moraiolo	2012		13
**at_14**	Azienda Mannucci Doandri DOP	Ceppeto (AR)	Blend	2011		14
**at_15**	Azienda "La Salceta"	Lorociuffenna (AR)	Frantoio, Leccino, Moraiolo	2012		15
**at_16c**	Tenuta "Montefoscoli "	Palaia (PI)	Frantoio, Leccino, Moraiolo	2012	Biologic oil	16
**at_18**	Azienda Terzi di Monte Oliveto	Asciano (SI)	Blend		Biologic oil	17
**at_19**	Frantoio Loziro	Murlo (SI)	Frantoio, Leccino, Moraiolo			18

**at_20**	Azienda Agricola Carraia	Podere Carraia, Petroio (SI)	Blend	2012		19
**at_21**	Tenuta Montalto	San Miniato (PI)	Frantoio, Leccino, Moraiolo	2012		20
**at_22**	Cosimo Maria Masina	San Miniato (PI)	Mignola	2012	Monocultivar	21
**at_23**	Tenuta San Quintino	San Miniato (PI)	Frantoio, Leccino, Moraiolo	2012		22
**at_24**	Azienda Olearea Chianti	Greve (FI)	Frantoio, Leccino, Moraiolo	2011		23
**at_25**	Azienda Agricola Donati	Casale Marittimo (PI)	Frantoio, Leccino, Moraiolo	2012		24
**at_26**	Piacenza Lucia Giole	Castagneto Carducci (LI)	Frantoio, Leccino, Moraiolo	2012		25
**at_27**	Villa Magra	Santa Luce (PI)	Frantoio, Leccino, Moraiolo	2012		26
**at_28**	Sopra Le Vigne	Calci (PI)	Frantoio, Leccino, Moraiolo	2012		27
**at_29**	Azienda Regionale Alberese	Alberese (GR)	Blend	2012	Biologic oil	28
---------	-------------	-------------	-- -	-------	----------------------- --	
**ap_1a**	Azienda Agricola Buondioli	Carpino (FG)	Frantoio	2012	Monocultivar	1

**ap_1b**	Azienda Agricola Buondioli	Carpino (FG)	Leccino	2012	Monocultivar	2
**ap_2c**	Azienda Agricola "D.Carbone"	Toritto (BA)	Blend	2012		3
**ap_3**	Antica Azienda Agricola Ricucci	Rodi Garganco (FG)	Blend	2012		4
**ap_4a**	Azienda Agricola "Cuonzo Franco"	Palombaio (BA)	Ogliarola	2012	Monocultivar	5
**ap_4b**	Azienda Agricola "Cuonzo Franco"	Palombaio (BA)	Coratina	2012	Monocultivar	6
**ap_5**	Masseria Chicco Rizzo	Martignano (LE)	Cellina di Nardò, Ogliarola	2012		7
**ap_6**	Cooperativa Agricola Olearia Sannicolese	Sannicola (LE)	Cellina di Nardò, Ogliarola	2012		8
**ap_7**	Tenuta Agricola "Serra Cicora"	Nardò (LE)	Blend	2012		9
**ap_8b**	Agrié di Nicola Santoro	Cursi (LE)	Cellina di Nardò, Ogliarola	2012		10
**ap_9a**	Conte	Sternatia (LE)	Coratina	2012	Monocultivar	11

**ap_9b**	Conte	Sternatia (LE)	Frantoio	2012	Monocultivar	12
**ap_9c**	Conte	Sternatia (LE)	Picholine	2012	Monocultivar	13
**ap_10**	Agrosì	Supersano (LE)	Blend	2012		14
**ap_11**	Terra del Sole	Rignano Garganico (FG)	Blend	2012		15
**ap_12a**	Adamo	Alliste (LE)	Cellina di Nardò, Ogliarola	2012		16
**ap_12b**	Adamo	Alliste (LE)	Cellina di Nardò, Ogliarola	2012		17
**ap_13a**	Agricola Nuova Generazione	Martano (LE)	Blend	2012	Mix green-ripe olives	18
**ap_13b**	Agricola Nuova Generazione	Martano (LE)	Blend	2012	Green olives	19
**ap_13c**	Agricola NuovaGenerazione	Martano (LE)	Blend	2012	mature	20
**ap_14a 3**	Agrinn	San Severo (FG)	Blend	2012	silos III	21
**ap_14b 1**	Agrinn	San Severo (FG)	Blend	2012	Biologic oil,silos 1	22
**ap_15**	FrantoioSciroppo	San Severo (FG)	Peranzana	2012	Monocultivar	23
**ap_16a**	Macchia del Barone	Melendugno (LE)	Blend	2012		24
**ap_16b**	Macchia del Barone	Melendugno (LE)	Blend	2012		25
**ap_16c**	Macchia del Barone	Melendugno (LE)	Blend	2012		26

**ap_17**	Bosco delleVergini	Bitonto (BA)	Cima di Bitonto	2012	Monocultivar	27
**ap_18**	Olio Colella	Corato (BA)	Coratina	2012	Monocultivar	28
**ap_19a**	Alea	Martano (LE)	Ogliarola	2012	Monocultivar	29
**ap_19b**	Alea	Martano (LE)	Cellina di Nardò, Ogliarola	2012		30
**ap_19c**	Alea	Martano (LE)	Cellina di Nardò	2012	Monocultivar	31

^{1}H NMR DOSY EXPERIMENTAL DETAILS:

2D cross-peaks are displayed, where the centres of the cross-peaks correspond to the calculated diffusion constant. An example of 2D DOSY experiment is shown in **Figure S1**.

Figure S1: Example of a 2D DOSY ^{1}H NMR plot resulting from the experiment on the EVOO sample (*ap_18*) acquired at T=298K.

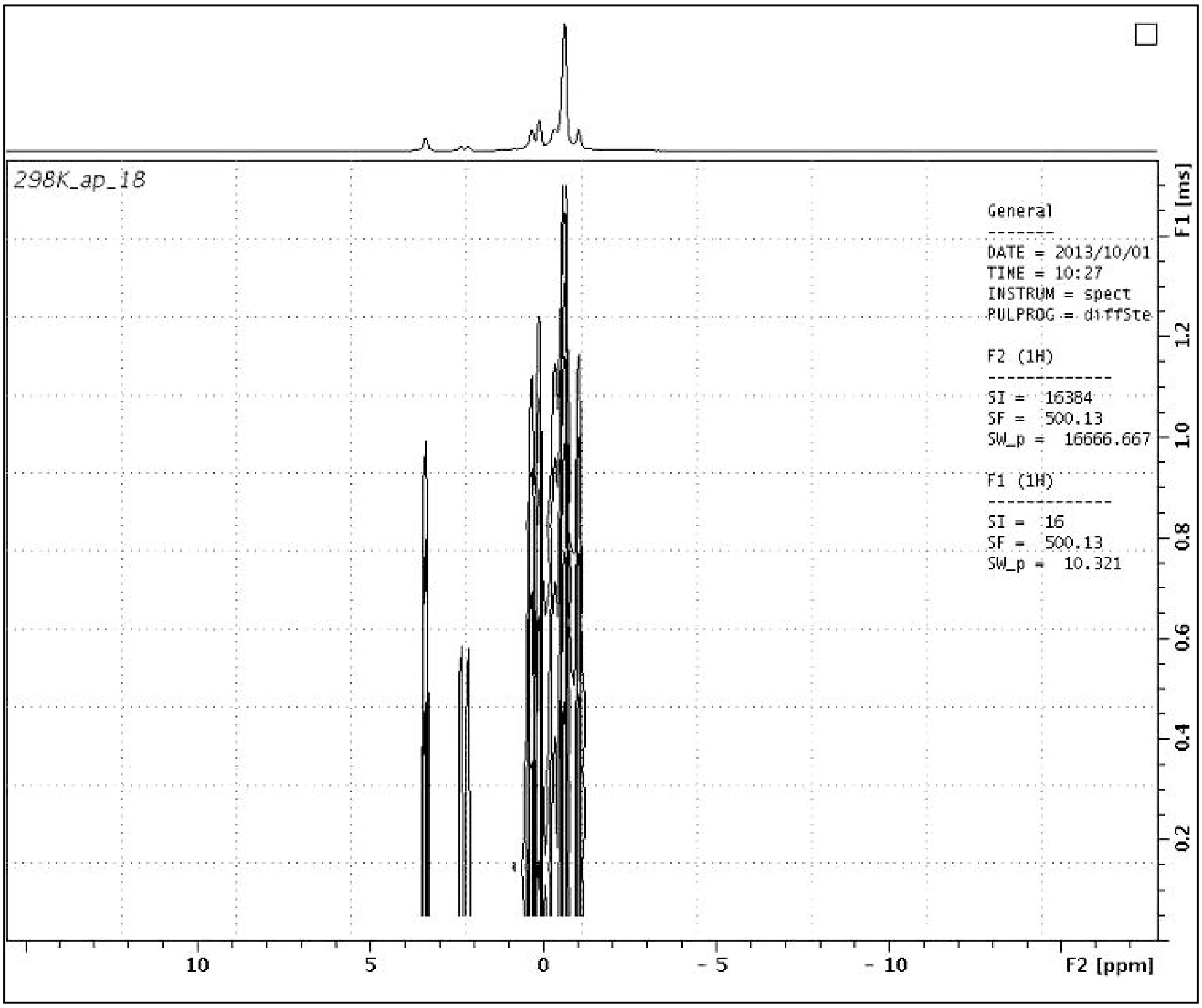

In ***Figure S2*** it is possible to see the ^{1}H NMR spectrum of the olive oil sample that shows the presence of nine peaks. Diffusion coefficients were calculated on four peaks indicated as 1, 3, 8 and 9.

Diffusion constant for the other peaks were not measured because their relatively low intensity under the optimised conditions for the applied NMR spin-echo sequence. In the main part of this research, for each oil sample, a single average value of diffusion coefficient, D, has been calculated as the average over the entire spectrum and these average values of the diffusion coefficient are reported and discussed in the Results and Discussion.

Figure S2: ^{1}H NMR spectrum of the olive oil (*ap_18*) acquired at T=298K at 500 MHz without any sample preparation.

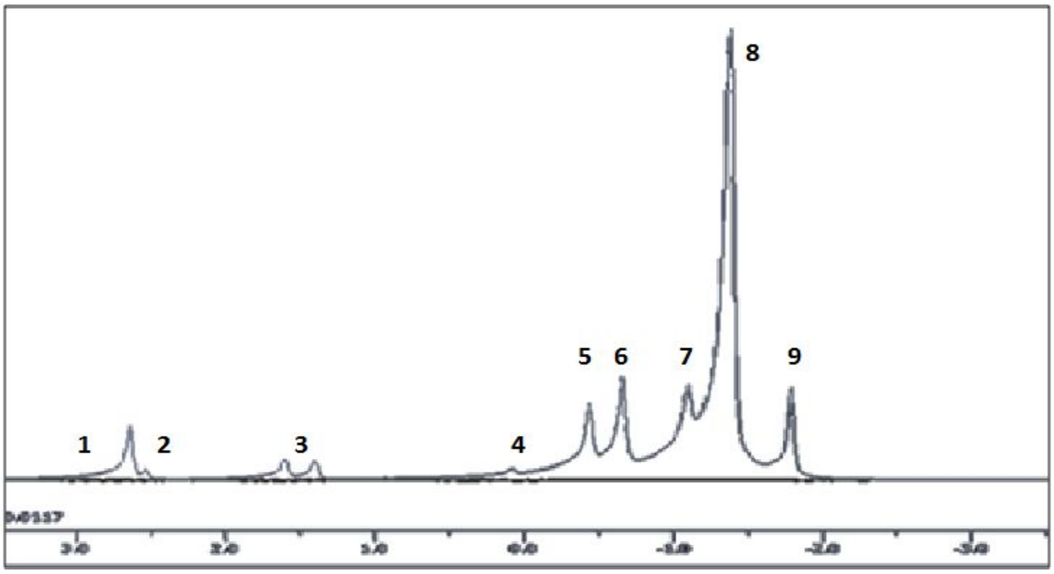

Temperature dependence of Diffusion coefficients in Olive oil and Sunflower oil.

The self-diffusion constant in oils is temperature-dependent. **Figure S3** shows the temperature dependence for two oils, EVOO ***ap_14a3*** and sunflower oil. As seen from the figure, both oils show similar temperature trends.

Figure S3: Temperature dependence of self-diffusion constant D for EVOO ***ap14_a3*** (see Table S1) and sunflower oil (SuO), measured by means of ^{1}H NMR DOSY technique. Dashed lines are data fits to an exponential function and serve as guides to an eye.

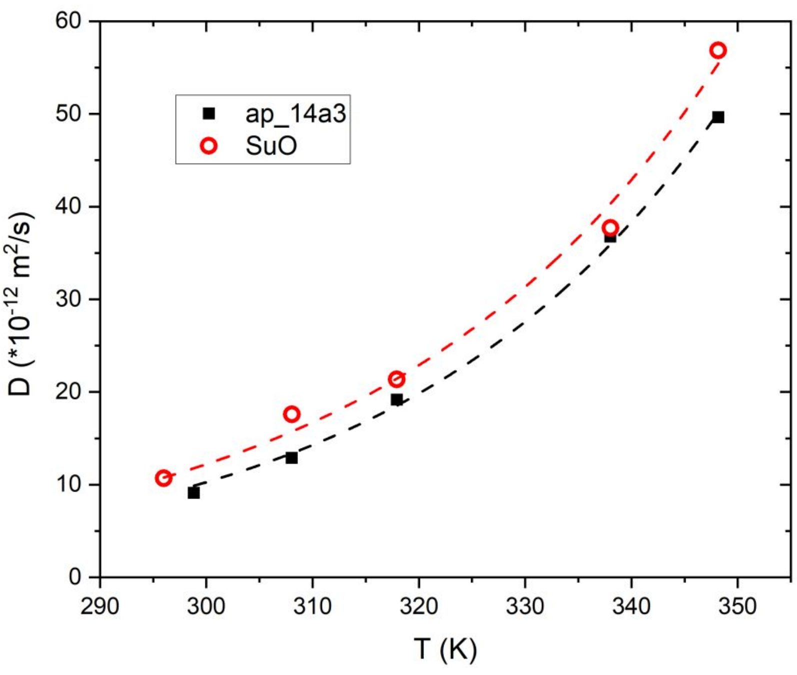

The self-diffusion constant is strictly connected with the viscosity parameter through the Stokes-Einstein equation. Assuming an Arrhenius-type relationship for viscosity, the temperature dependence of diffusion constant can be expressed as:

$$D = D_0 \cdot e^{-\frac{E_a}{RT}} \qquad \text{(S1)}$$

The values of activation energy of the self-diffusion process in the analysed oils could be estimated from **Figure S3** (E_a~30.3 kJ/mol for EVOO and E_a~26.4 kJ/mol for SuO). These values are in agreement with data found in the literature (A. Rachocki & J. Tritt-Goc, 2014).
